# Complete Recovery from COVID-19 Bilateral Pneumonia in an Immunosuppressed Man with Immune-Mediated Necrotizing Myopathy

**DOI:** 10.1155/2020/8886324

**Published:** 2020-10-21

**Authors:** Thomas C. Bolig, Nada Abdulaziz, Elena Schiopu

**Affiliations:** ^1^Department of Internal Medicine, University of Michigan, 1500 E Medical Ctr Dr, Ann Arbor, MI 48109, USA; ^2^Department of Rheumatology, University of Michigan, 1500 E Medical Ctr Dr, Ann Arbor, MI 48109, USA

## Abstract

Immune-mediated necrotizing myopathy (IMNM) is a rare form of idiopathic immune myopathy (IIM) that requires immunotherapies, including immunosuppressive medications, if severe. There is a paucity of data regarding outcomes of patients with immune-mediated polymyositis who continue immunosuppressive medications during the COVID-19 pandemic. This is the first reported case of COVID-19 in a patient with IMNM. Despite being on two immunotherapies, having risk factors, and having radiographic abnormalities on chest X-ray, the patient had an unremarkable COVID-19 course. He was discharged from the emergency department with a 7-day course of azithromycin and quickly resumed his immunotherapies, but he experienced a flare in his myositis. The 14-week follow-up computed tomography (CT) was negative for residual pneumonitis or fibrosis. More data are needed regarding management and prognosis of patients with connective tissue diseases who become infected with SARS-CoV-2.

## 1. Introduction

Immune-mediated necrotizing myopathy (IMNM) is a recently recognized inflammatory myositis characterized by proximal muscle weakness and rare extra-muscular involvement. It comprises 20 percent of the 2–7 per million yearly cases of idiopathic inflammatory myopathies [1]. IMNM can be differentiated from statin-induced myopathy since symptoms of myositis persist after the withdrawal of statin therapy and usually the anti-HMG-CoA reductase antibody is negative [2]. There is a subset of statin-induced IMNM in which the anti-HMG-CoA reductase antibody is positive and a statin-induced myopathy will have upregulation of sarcolemmal MHC class I on muscle biopsy, which is typically absent in IMNM [3]. The defining pathologic features are myofiber necrosis and minimal inflammatory cell infiltrate [4]. Immune-mediated necrotizing myopathy requires treatment with immunosuppressive medications if severe, which is more frequently associated with the presence of anti-signal recognition particle (SRP) antibodies [5]. A severe presentation of IMNM may only respond to aggressive immunosuppressive medications. During the COVID-19 pandemic, the continuation of immunosuppressants for the panoply of connective tissue diseases has been an area of ongoing debate, as the risk of severe COVID-19 has to be balanced with the risk of flares (potentially requiring high-dose glucocorticoids to manage). Little is known regarding outcomes of patients with IIMs who contract COVID-19 while receiving immunosuppressive therapy. We present the first reported case of COVID-19 in a patient with IMNM.

### 1.1. Case Presentation

A 54-year-old white man with a history of immune-mediated necrotizing myopathy and obesity (body mass index (BMI) of 35) presented to the emergency department (ED) with five days of fevers (102–104 degrees Fahrenheit), chills, myalgia, and dry cough. His IMNM was diagnosed 1.5 years prior to this ED presentation. The diagnosis was based on rapidly progressive symmetrical proximal muscle weakness; laboratory tests demonstrated an elevated aldolase (75 IU/L; reference range 1–7 IU/L) and creatine kinase (CK) (5312 IU/L; reference range 38–240 IU/L), a low titer anti-mitochondrial antibody (1 : 80), an anti-SSA 52 Kd of 24 (reference range <20 units), and a muscle biopsy showing pauci-immune myositis. His biopsy demonstrated an upregulation of MHC1, arguing against IMNM; however, desmin, C5b9, TDP43, Cd3/SMA, and CD163/8 immunostaining confirmed scattered muscle fiber necrosis, myophagocytosis, and degenerating-regenerating fibers consistent with IMNM. CD45 and CD68 immunostaining was not performed. The patient's anti-SRP and anti-HMG-CoA reductase antibodies were notably negative, and he had no prior exposure to a statin. His IMNM had been successfully treated with mycophenolate mofetil (MMF, 3 g/day) and intravenous immunoglobulins (IVIG, Gammagard 2 g/kg/month).

**Figure 1:**
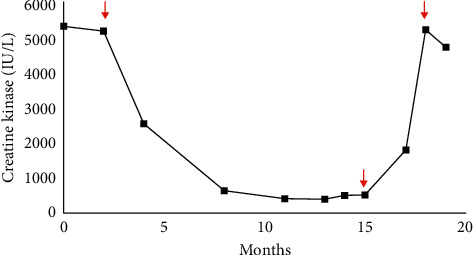
Trends in serum creatine kinase (IU/L) from IMNM diagnosis through his course of COVID-19. First arrow: initiation of concomitant IVIG and mycophenolate mofetil. Second arrow (15 month): the patient's immunotherapies were held, resulting in a rapid return of creatine kinase to levels greater than 5000 IU/L. Third arrow: the patient restarted IVIG and mycophenolate mofetil.

**Figure 2:**
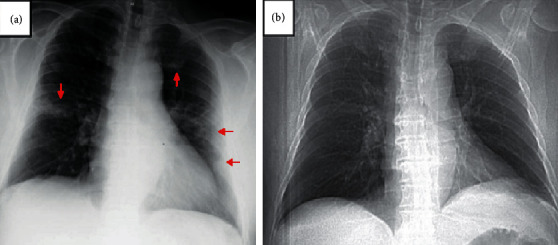
Chest radiographs of the patient. (a) Chest radiograph on presentation to the emergency department; the arrows delineate areas of patchy opacities that tend to favor the lung periphery. (b) Scout film from a previous high-resolution chest CT approximately 1.5 years prior to his COVID-19 presentation for comparison.

**Figure 3:**
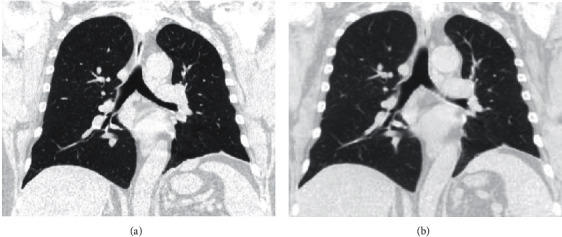
Comparison of coronal images from noncontrast, high-resolution chest computed tomography (CT) before and after COVID-19 pneumonia. (a) demonstrates a coronal slice of the patient's high-resolution chest CT 1.5 years prior to presentation. (b) is a follow-up chest CT 3 months after the patient's recovery from COVID-19 that shows absence of residual pneumonitis or new fibrosis.

When we were notified of the high fever, we instructed the patient to stop taking MMF (Figure 1). Initial rapid strep and flu swabs were negative, and a SARS-CoV-2 real- time polymerase chain reaction (RT-PCR) nasopharyngeal swab was positive, prompting him to visit the emergency department. In the ED, his vital signs were stable including 99% on pulse oximetry on room air. No objective fever was recorded during his ED visit. He reported fevers, chills, myalgia, dry cough, and shortness of breath. He did not have chest pain, nausea, vomiting, diarrhea, abdominal pain, or lower extremity edema. His labs were significant for leukopenia (3.62 K/mcl; reference range 4.0–10.0 K/uL) without lymphopenia (absolute lymphocyte count 1.25 K/mcL; reference range 1.2–4.0 K/uL). His renal function was normal (creatinine 0.93 mg/dL; reference range 0.70-1.30 mg/dL) with a NT pro-BNP of <50 pg/mL (reference range 50–137 pg/mL) and an unremarkable procalcitonin of 0.12 ng/mL (reference range <0.10 ng/mL). His chest X-ray demonstrated multiple patchy opacities in the periphery of both lungs (Figure 2(a)). His IMNM was not previously associated with structural heart or lung disease, and former chest CT was unremarkable a year prior to the current presentation (Figure 3(a)). As part of his myositis workup, he had a normal transthoracic echocardiogram the year prior to presentation.

The ED physician monitored him and, due to the patient's clinical stability, discharged him home with a seven-day course of azithromycin (500 mg day 1 and then 250 mg daily for 6 more days) and CDC instructions for self-quarantine. The rest of his COVID-19 course was unremarkable. He did not have a recorded fever after initiation of azithromycin and his cough and myalgia resolved 3 days afterward. The patient resumed MMF 6 weeks since first symptoms and monthly infusions of IVIG 8 weeks since first symptoms without recurrence of respiratory symptoms. The patient did not resume IVIG immediately due to his reluctance to return to the infusion center since he thought he may have been infected with SARS-CoV-2 during his last IVIG infusion.

Unfortunately, his IMNM disease flared (Figure 1) due to weeks of immunosuppression withdrawal. Despite re-initiating MMF and IVIG, he had a tedious recovery and remained weak. This flare could have been myositis secondary to COVID-19, although it would have been a delayed manifestation of his otherwise unremarkable course. The timing seemed more consistent with cessation of his MMF and IVIG (Figure 1). Three months after the initial infection confirmation, we obtained an additional high-resolution CT of the chest (Figure 2(b)) which showed complete resolution of the interstitial infiltrates and no new areas of fibrosis.

## 2. Discussion

To our knowledge, this is the first reported case of COVID-19 in IMNM. Despite a relatively benign course, it is notable that he had multiple risk factors for progression to severe COVID: male sex, obesity, immunosuppressive medication, and an abnormal chest X-ray [6]. His chest X-ray included multiple patchy, airspace opacities in the periphery of both lungs. These findings on the abnormal chest X-ray are indicative of alveolar filling, and the pattern of favoring the lung periphery is commonly described in COVID-19 [7]. The variation in radiographic findings with severity of disease coupled with a difficult to predict course in COVID-19 makes triaging and management of patients with connective tissue disease particularly challenging. In this case, despite no overt evidence of bacterial or atypical pneumonia (i.e., low procalcitonin, negative respiratory panel, and positive SARS-CoV-2 RT-PCR), the patient was treated with azithromycin. He also resumed his IVIG and mycophenolate mofetil rather quickly without a major complication.

### 2.1. COVID-Related Extrapulmonary Manifestations, including Myositis

Since the beginning of the pandemic, multiple studies established that COVID-19 is a truly systemic disorder [8]: pulmonary, cardiovascular, hepatobiliary, gastrointestinal, renal, neurological, and musculoskeletal. Myalgia is present in up to 36% of the COVID-19 patients, and 16–33% have elevated CK levels, and patients with myalgias have a higher chance of having abnormal lung images [6, 9]. We found 2 cases of COVID-19-related inflammatory myopathies. The first case involved a 58-year-old female presenting with progressive proximal, facial, and bulbar muscle weakness, elevated CK, and a muscle biopsy showing perivascular inflammatory infiltration with endomysial extension, regenerating fibers, and upregulation of human leukocyte antigen class ABC expression on nonnecrotic fibers who had improvement in muscle pain and weakness after 5 days of 1000 mg of methylprednisolone [10]. The second case was a male patient with rapidly progressive proximal muscle weakness, an elevated CK, magnetic resonance imaging (MRI) supportive of inflammatory myositis, and pneumonitis who required ICU admission [11].

We then searched the current literature for any reports of patients with pre-existing IIM and COVID-19. A case series of 4 patients with rheumatological disorders from Malaysia described an 80-year-old female with polymyositis on azathioprine as having a benign course [12]. No additional data aside from the presence of concomitant diabetes mellitus were provided.

### 2.2. Pneumonitis Reversibility in COVID-19

Ongoing discussion regarding the potential of COVID-19 pneumonitis to evolve into fibrotic lung disease [8] exists, particularly since there are similarities between the major risk factors for severe COVID-19 and those for idiopathic pulmonary fibrosis (IPF): older age, male sex, hypertension, and diabetes [13]. Additionally, we learned from previous coronavirus infections such as severe acute respiratory syndrome (SARS) and Middle East respiratory syndrome (MERS) that there could be substantial fibrotic consequences [14]. Although too early to comment on the proportion of COVID-19 patients who go on to develop pulmonary fibrosis, a study analyzing pulmonary function data from discharged patients showed 47% of the 108 patients had impaired gas transfer and 27 (25%) had reduced total lung capacity [15], which was much worse in patients with severe disease.

One of the most important clinical manifestations of COVID-19 pneumonitis is acute respiratory distress syndrome (ARDS); although many patients who develop ARDS survive, elevated interleukin (IL-1*β* and IL-6) levels predict progression to lung fibrosis and death. Many survivors have significantly impaired quality of life, exercise capacity, and even CT abnormalities following ARDS [15, 16]. The ARDS data could be easily extrapolated to post-COVID-19 clinical recovery. Our patient, however, had no evidence of residual pneumonitis or pulmonary fibrosis.

### 2.3. COVID-19 Treatment in Rheumatic Disorders

The American College of Rheumatology COVID-19 guidelines suggest restarting DMARDs within 7–14 days from resolution of symptoms, or 10–17 days from a positive SARS-CoV-2 RT-PCR test [17]. We decided to wait 6 weeks from the positive RT-PCR for this patient, which led to a disease flare. There remains controversy about continuing MMF during COVID-19 active infection.

The management of patients' immunotherapies in connective tissue disease during the COVID-19 era is still being investigated. Our patient was on two immunotherapy medications for suppression of his severe IMNM. MMF is a broad immunosuppressant with activity against T- and B-cells making individuals more susceptible to bacterial and viral infections, but it interestingly demonstrates some *in vitro* and *in vivo* activity against several viruses [18]. The blood product IVIG is not immunosuppressive and has numerous applications and a number of adverse effects, and aside from autoimmune disorders, it has been used in severe viral pneumonias with some efficacy [19]. There is no evidence to suggest that it is beneficial against COVID-19. Clinical trials using both regular IVIG and convalescent plasma are currently ongoing. A trial using convalescent plasma as an adjunct was a negative study but was associated with a negative conversion rate of viral PCR at 72 hours [20]. Not only is more information needed regarding the management of immunosuppressive medications but there is also growing knowledge of outcomes of individuals with COVID-19 in polymyositis and other connective tissue diseases.

## 3. Conclusion

A middle-aged obese male with a history of immune-mediated necrotizing myopathy on mycophenolate mofetil and IVIG had a complete recovery from COVID-19 pneumonia despite receiving immunosuppressive medications and risk factors for progression to severe disease. He completed a course of azithromycin and resumed his immunosuppressive medications without COVID-19 symptom recurrence or lung damage. His underlying IMNM flared (Figure 1) due to the 6-week hold of MMF and 8-week hold of IVIG which supports the current discussion that immunosuppression could be continued during selected COVID-19 infections. This is the first known reported case of COVID-19 in IMNM. There is still much to learn regarding prognosis and management of COVID-19 in patients with connective tissue disease on immunosuppressive medications.

## Data Availability

The underlying data of this case report are located in the University of Michigan Health System electronic medical record.
